# One‐stage individual participant data meta‐analysis models: estimation of treatment‐covariate interactions must avoid ecological bias by separating out within‐trial and across‐trial information

**DOI:** 10.1002/sim.7171

**Published:** 2016-12-01

**Authors:** Hairui Hua, Danielle L. Burke, Michael J. Crowther, Joie Ensor, Catrin Tudur Smith, Richard D. Riley

**Affiliations:** ^1^ Biostatistics & Data Sciences AsiaBoehringer IngelheimShanghai200040China; ^2^ Research Institute for Primary Care and Health SciencesKeele UniversityKeeleStaffordshireST5 5BGU.K.; ^3^ Department of Health SciencesUniversity of LeicesterLeicesterLE1 7RHU.K.; ^4^ Department of Medical Epidemiology and BiostatisticsKarolinska InstitutetS‐171 77StockholmSweden; ^5^ MRC North West Hub for Trials Methodology Research, Department of BiostatisticsUniversity of LiverpoolLiverpoolL69 3GLU.K.

**Keywords:** ecological bias, effect modifier, meta‐analysis, stratified/precision medicine, treatment‐covariate interaction

## Abstract

Stratified medicine utilizes individual‐level covariates that are associated with a differential treatment effect, also known as treatment‐covariate interactions. When multiple trials are available, meta‐analysis is used to help detect true treatment‐covariate interactions by combining their data. Meta‐regression of trial‐level information is prone to low power and ecological bias, and therefore, individual participant data (IPD) meta‐analyses are preferable to examine interactions utilizing individual‐level information. However, one‐stage IPD models are often wrongly specified, such that interactions are based on amalgamating within‐ and across‐trial information. We compare, through simulations and an applied example, fixed‐effect and random‐effects models for a one‐stage IPD meta‐analysis of time‐to‐event data where the goal is to estimate a treatment‐covariate interaction. We show that it is crucial to centre patient‐level covariates by their mean value in each trial, in order to separate out within‐trial and across‐trial information. Otherwise, bias and coverage of interaction estimates may be adversely affected, leading to potentially erroneous conclusions driven by ecological bias. We revisit an IPD meta‐analysis of five epilepsy trials and examine age as a treatment effect modifier. The interaction is −0.011 (95% CI: −0.019 to −0.003; *p* = 0.004), and thus highly significant, when amalgamating within‐trial and across‐trial information. However, when separating within‐trial from across‐trial information, the interaction is −0.007 (95% CI: −0.019 to 0.005; *p* = 0.22), and thus its magnitude and statistical significance are greatly reduced. We recommend that meta‐analysts should only use within‐trial information to examine individual predictors of treatment effect and that one‐stage IPD models should separate within‐trial from across‐trial information to avoid ecological bias. © 2016 The Authors. *Statistics in Medicine* published by John Wiley & Sons Ltd.

## 1 Introduction

There is an increasing interest in personalized or stratified medicine, where the aim is to tailor treatments to individuals or to groups of similar individuals based on their particular characteristics 1. This allows clinicians to optimize treatment decisions and reduce unnecessary costs, in order to select treatments for individual patients that are most likely to benefit (or least likely to harm) them. For example, trastuzumab is only given to the subgroup (stratum) of breast cancer patients who are human epidermal growth factor receptor 2 (HER‐2) positive, as it is known to lock on to the HER‐2 protein, block the receptor and stop the cells from dividing and growing 2. It is therefore considered unnecessary for those who are HER‐2 negative.

A key component of stratified medicine research is to identify individual‐level characteristics (covariates) that are associated with a differential treatment effect 1. These are referred to as *treatment‐covariate interactions* in this article, but other names include effect‐modifiers, moderators, subgroup effects and predictive markers. Although some treatment‐covariate interactions, such as HER‐2, are suspected in advance because of strong biological rationale, others are only identified following secondary investigations of existing data. A single randomized trial tends to have low power to detect treatment‐covariate interactions because, usually, they are powered on the overall treatment effect in the population of interest 3. However, when multiple trials are available, meta‐analysis provides the opportunity to increase power to detect true treatment‐covariate interactions by combining their data 4.

In aggregate data meta‐analysis, where aggregated study results are obtained and then synthesized, treatment‐covariate interactions are usually investigated using meta‐regression 5, which quantifies the across‐studies association between the overall treatment effect and aggregated *trial‐level* covariates (such as the mean age of participants, or the proportion of participants that are male). However, this approach usually has low power to identify genuine treatment effect modifiers at the individual‐level because of the usually small number of studies in meta‐analysis 6; there needs to be large variation in the aggregated covariate values across trials for the power to be feasible 7. Further, it is also prone to study‐level confounding and ‘ecological bias’ 8, which means the observed across‐study relationships do not properly reflect the individual‐level relationships within trials. For example, meta‐regression may identify that studies containing a larger proportion of male participants have a larger overall treatment effect; however, this may be due to such studies also having a higher dose of the treatment, and therefore, improved effect is due to the dose and not being male rather than female 9.

Individual participant data (IPD) meta‐analysis can overcome the issues of low power and potential ecological bias by examining within‐study interactions at the individual‐level (rather than across‐study interactions at the trial‐level). Here, the participant‐level data are analysed in either a two‐stage or a one‐stage approach in order to summarize the interaction between treatment effect and individual covariates 10, 11, 12, 13. The two‐stage approach is the most straightforward, where firstly the treatment‐covariate interactions are estimated in each trial separately, and then secondly, these are pooled using a traditional (e.g. inverse‐variance weighted) meta‐analysis model. By only pooling within‐study information, this approach automatically avoids ecological bias 12, 14; however, the second stage requires one to assume study estimates are approximately normally distributed and that their estimated variances are known, which is contentious when included studies only have small number of patients and/or events 15, 16.

The alternative one‐stage approach analyses all patient‐level data from every trial in one step whilst accounting for the clustering of patients within studies using a hierarchical model 17, 18, 19. In contrast to the two‐stage approach, one‐stage meta‐analysis models allow a more exact likelihood to be specified and automatically account for the correlations amongst parameters 17. However, when investigating treatment‐covariate interactions, it has been shown that the one‐stage approach does not automatically avoid ecological bias when estimating treatment‐covariate interactions; that is, estimation of interaction terms in a one‐stage meta‐analysis might merge (amalgamate) both within‐trial and across‐trial information 9, 20, 21. To avoid potential ecological bias, one needs to separate out within‐trial and across‐trial interaction effects in the model specification 9, 12, 14, 22, 23, which is also recognized in areas outside the meta‐analysis field that contains clustering 24, 25, 26, 27.

Though this topic has been previously discussed in the meta‐analysis literature, our recent experience is that the issue of ecological bias is still being ignored in many applied one‐stage IPD meta‐analyses, especially in the context of time‐to‐event outcomes. For example, in 2015, Sahgal *et al.* perform a one‐stage IPD meta‐analysis of randomized trials evaluating stereotactic radiosurgery with or without whole‐brain radiation therapy for patients presenting with one to four brain metastases 28. They conclude that ‘age was a significant effect modifier (*p* = 0.04) favouring stereotactic radiosurgery alone in patients ≤50 years of age’. However, the publication does not state that ecological bias was considered or that within‐study and across‐study interactions were separated.

Data sharing is becoming expected in medical research 29, and the number of IPD meta‐analyses is rising 11, 30, many of which aim to identify treatment effect modifiers. New protocols are being published each month for IPD meta‐analyses, which pre‐define their statistical analysis plan. For example, van Middelkoop *et al.* 31 provide a protocol for their IPD meta‐analysis of trials investigating the effectiveness of intra‐articular glucocorticoid injections in patients with knee or hip osteoarthritis. The authors state they will use a one‐stage model, and to examine how pain or inflammation modify treatment effect, they will include ‘an interaction term (pain × treatment or inflammation × treatment)’; however, perhaps unknowingly, this will amalgamate within‐study and across‐study interactions.

A strong message is thus urgently needed: researchers should avoid potential ecological bias in their one‐stage IPD meta‐analyses. The aim of this article is to show how this can be achieved and to illustrate the consequences of ignoring it through a detailed simulation study and an illustrated example for time‐to‐event outcomes. In particular, we extend the one‐stage framework of Tudur Smith *et al*.^32^ proposed in this journal, who showed how to examine treatment‐covariate interactions but did not adjust for ecological bias. Other recent IPD meta‐analysis articles of time‐to‐event data consider mainly two‐stage methods 14, 33, 34, evaluate or compare one‐stage and two‐stage analyses for the overall treatment effect 35, 36, or focus on estimation techniques 37, 38, 39, including parametric approaches 40, for modelling baseline risks and overall effects. However, our main focus is on how to appropriately estimate treatment‐covariate interactions in this context. Fisher *et al*. provide an excellent overview of methods for estimating interactions in meta‐analysis 12, with illustration including survival examples; however, our work extends this through the detailed simulation study across a wide range of scenarios, with a novel example in epilepsy.

The remainder of the article is as follows. Section 2 introduces four key fixed‐effect and random‐effects Cox regression models that can be used to investigate treatment‐covariate interactions. Section 3 details the methods and results of the simulation study, which includes scenarios for both binary and continuous covariates, with and without trial‐level confounders. The key findings are then illustrated in the context of a real IPD meta‐analysis dataset in Section 4, and Section 5 concludes with some discussion.

## 2 Estimation of treatment‐covariate interactions in a one‐stage IPD meta‐analysis models for time‐to‐event data

Consider the IPD meta‐analysis of time‐to‐event data across *j* = 1 to *J* trials. Let *x_ij_* be a participant‐level covariate of interest, which can be continuous such as age, or binary such as sex, and let *z_ij_* denote whether the *i*th patient in the *j*th trial is in the treatment group or in the control group (1 = treatment group, 0 = Control group). For each patient, we also have whether they had the event or were censored and their event or censoring time. We now introduce four key specifications of a one‐stage IPD meta‐analysis model, based on Cox proportional hazards models. These all specify a *separate* baseline hazard per trial (i.e. not necessarily proportional), assume a constant treatment effect over time in each trial (i.e. hazard rates for the treatment and control groups are assumed proportional), and either merge or separate within‐study and across‐study treatment‐covariate interactions. Of course, other specifications are possible (e.g. proportional baseline hazards across trials); however, here, the main focus is on the specification of the interactions. For a comprehensive introduction of the framework of (random‐effects) models for Cox regression and meta‐analysis of time‐to‐event data, we refer the reader elsewhere 32, 35, 36, 37, 38, 39, 41.

### 2.1 Merging within‐study and across‐study interactions

A simple, but potentially naïve, model that ignores any residual between‐study heterogeneity and amalgamates within‐trial and across‐trial interactions can be written as follows:

(1) $$\lambda_{ij}\left(t\right)=\lambda_{0j}\left(t\right)\exp\left(\beta_{1}z_{ij}+\beta_{2j}x_{ij}+\beta_{T}x_{ij}z_{ij}\right)$$

Here, *λ*_0*j*_(*t*) denotes the unique baseline hazard function in the *j*th trial, and *x*_*ij*_ *z*_*ij*_ represents the interaction term between the treatment and covariate of interest, which is an amalgamation of within‐study and between‐study information. The constant coefficient *β*_1_ is the treatment effect (i.e. the change in the log hazard for patients in the treatment group rather than control group) where *x*_*ij*_ = 0, *β*_2*j*_ is the study‐specific change in the log hazard for a one‐unit increase in the patient‐level covariate where *z*_*ij*_ = 0, and *β*_*T*_ denotes the additional change in the log hazard for patients in the treatment group compared with the control group for one unit increasing values of *x*_*ij*_. We note that the separate baseline hazard per trial (*λ*_0*j*_(*t*)) is essential to account for clustering of patients within trials 42. A separate adjustment term (*β*_2*j*_) is also ideally preferred, as the effect of the covariate may also differ across trials; however, this also increases the number of parameters to estimate, and so, when there are non‐convergence issues, it may be necessary to make a stronger assumption that this adjustment term is the same in each trial.

We could also allow for residual heterogeneity in the treatment effect (i.e. not explained by the interaction term), in a random‐effects model:

(2) $$\begin{array}{l} \lambda_{ij}\left(t\right)=\lambda_{0j}\left(t\right)\exp\left(\beta_{1j}z_{ij}+\beta_{2j}x_{ij}+\beta_{T}x_{ij}z_{ij}\right)\\ \beta_{1j}=\beta_{1}+b_{1j}\\ b_{1j}\sim N\left(0,\tau^{2}\right) \end{array}$$

The coefficient *β*_1_ is now the average log hazard ratio for a distribution of possible treatment effects where *x*_*ij*_ = 0 and the random variable *b*_1*j*_ follows a *N*(0, *τ* ^2^) distribution, where *τ* ^2^ is the residual between‐trial heterogeneity. One could also include a random effect on the interaction term.

### 2.2 Separating within‐study and across‐study interactions

When we include the interaction as in (1) and (2), it amalgamates within‐ and across‐trial interactions. Alternatively, we can model these separately by centring the covariate *x*_*ij*_ about the mean, ${\bar{x}}_{j}$, in each trial *j* and also including the mean ${\bar{x}}_{j}$ as an additional adjustment term to explain between‐study heterogeneity. For example, if we assume there is no residual between‐study heterogeneity in the treatment effect after including the covariate mean, ${\bar{x}}_{j}$, then we can extend fixed‐effect model (1) to

(3) $$\begin{array}{l} \lambda_{ij}\left(t\right)=\lambda_{0j}\left(t\right)\exp\left(\beta_{1j}z_{ij}+\beta_{2j}x_{ij}+\beta_{W}\left(x_{ij}-{\bar{x}}_{j}\right)z_{ij}\right)\\ \beta_{1j}=\alpha +\beta_{A}{\bar{x}}_{j} \end{array}$$

Allowing for residual between‐study heterogeneity, we can extend model (2) to

(4) $$\begin{array}{l} \lambda_{ij}\left(t\right)=\lambda_{0j}\left(t\right)\exp\left(\beta_{1j}z_{ij}+\beta_{2j}x_{ij}+\beta_{W}\left(x_{ij}-{\bar{x}}_{j}\right)z_{ij}\right)\\ \beta_{1j}=\alpha +\beta_{A}{\bar{x}}_{j}+b_{1j}\\ b_{1j}\sim N\left(0,\tau^{2}\right) \end{array}$$

Parameters in models (3) and (4) are as discussed before, but additionally, the within‐trial coefficient, *β*_*W*_, denotes the expected change in the treatment effect (log hazard rate ratio for individuals who receive the treatment compared with control) for each one unit increase in *x*_*ij*_, and the across‐trial coefficient, *β*_*A*_, denotes the expected change in the overall study treatment effect (log hazard rate ratio) for every one unit increase in ${\bar{x}}_{j}$.

Centring the patient‐level covariate in models (3) and (4) ensures that *β*_*W*_ now only explains within‐study variability, and *β*_*A*_ only explains between‐study variability. In other words, the within‐ and the across‐trial interaction estimates are now uncorrelated with each other and thus disentangled 9, 26. In contrast, models (1) and (2) provide some weighted average of *β*_*W*_ and *β*_*A*_, which will increase power but at the expense of potential ecological bias. Models (3) and (4) also allow one to estimate the magnitude of ecological bias by *β*_*W*_–*β*_*A*_ 20, although there will usually be low power to identify, or statistically test, for ecological bias using this approach because of typically imprecise estimates of *β*_*A*_. For researchers who prefer not to explain between‐study heterogeneity, then model (4) can be fitted without the $\beta_{A}{\bar{x}}_{j}$ term, and the interpretation of *β*_*W*_ would remain intact.

### 2.3 Applicability of *β*_*W*_ and *β*_*A*_

In this paper, the key focus is on providing interaction estimates that are meaningful to stratified (personalized) medicine, so that treatment decisions can be tailored to individuals based on their covariate values. For this reason, the main parameter of interest from the previous models is *β*_*W*_ because this explains differences in treatment response at the individual‐level and thus reduces within‐trial (patient‐level) variability. In contrast, *β*_*A*_ explains differences in population average treatment effects. Although this is helpful to reduce between‐study variability, and perhaps to inform population‐level comparisons (or predictions) of overall treatment effects (or overall prognosis 43), it is potentially misleading to use *β*_*A*_ to make inferences about individuals. This is demonstrated in detail in the simulations and examples in Sections 3 and 4, where we compare estimates for *β*_*W*_ and *β*_*A*_, and also their amalgamation (*β*_*T*_), in a range of settings.

### 2.4 Model estimation

To fit the stratified Cox regression for models (1) and (3), many standard statistical packages are available, such as *coxph* in R 44 and *stcox* in Stata 45, which maximize the profile likelihood. To estimate the random‐effects models (2) and (4), a package such as *coxme* in R could be utilized, for example where the random‐effects are integrated out to maximize the integrated partial likelihood 46. Crowther *et al*. also show how to fit models (3) and (4) using Poisson regression with maximum likelihood via Gauss–Hermite quadrature 37, which has the advantage of also providing an estimate of the baseline hazards (one for each trial) if necessary (for example, for absolute risk predictions).

## 3 Simulation study to evaluate treatment‐covariate interactions

We now describe two simulation studies to assess the performance of the models with merged (i.e. (1) or (2)) or separated interaction terms (i.e. (3) or (4)), when either ignoring ((1) and (3)) or accounting for ((2) and (4)) residual between‐study heterogeneity. In the first simulation study, we exclude any trial‐level confounding factor (‘No confounding’ simulation study). In the second simulation study, we include a confounding factor (‘Confounding’ simulation study). In each simulation study, we consider binary (sex) or continuous (age) variables and their interaction with treatment. The *survsim* package in Stata is utilized to simulate survival data 47, and the main steps of the simulation study are summarized as follows 48:

Step 1 Each simulated IPD meta‐analysis dataset consists of *J* trials, with *J* fixed per simulation scenario. The number of patients in each trial was randomly determined by sampling from a normal distribution with mean *N* and standard error, *N/*5, where *N* is fixed per simulation scenario.
Step 2 In each individual trial, each patient has an equal chance to be assigned to the experimental (treatment) group *z_ij_* = 1 or the control group *z_ij_* = 0 by randomly sampling from a Bernoulli (0.5) distribution.
Step 3a If the covariate *x* is the binary variable, such as sex (1 = Male, 0 = Female), then for the *i*th patient in the *j*th trial, we firstly sample a mean *(x_j_)* in the *j*th trial from a uniform distribution (0*.*5 − *V*_1_ *,* 0*.*5 + *V*_1_) where *V*_1_ is chosen to be between 0 and 0.5 and then randomly sample *x_ij_* for each patient in each study from a Bernoulli distribution with the obtained mean *x_j_*.

If the covariate *x* is the continuous variable, age, then for the *i*th patient in the *j*th trial, a mean *(x_j_)* in the *j*th trial is firstly sampled from a uniform distribution (50 − *V*_1_ *,* 50+ *V*_1_) where *V*_1_ is chosen to be between 0 and 35 and then *x_ij_* is sampled from a normal distribution truncated at 15 and 85 with the obtained mean *x_j_* and a standard error *V*_2_, where *V*_2_ is chosen to be a positive number. *V*_1_ and *V*_2_ are fixed per simulations scenario.

It is important to note that *V*_1_ defines the amount of across‐trial variability in the mean covariate values, whereas *V*_2_ defines the amount of within‐trial variability in the individual covariate values. If *V*_1_ is large then there is a greater spread of trial‐level mean covariate values, and thus there is more opportunity (greater power) for any across‐trials information to contribute in subsequent one‐stage meta‐analyses 7.

Step 3b In addition, for simulation scenarios with study‐level confounding, we define *y_j_* to indicate whether the *j*th clinical trial has a higher dose of the treatment in the experimental arm (1 = yes, 0 = no). All trials with the mean of the binary covariate (sex) above 0.5 or the mean of continuous covariate (age) above 50 are given this extra effect *β*_4_ (*y_j_* = 1).
Step 4 In each study separately, we use the *survsim* package in Stata to generate the patient‐level survival data (that is event times) for the ‘no confounding’ simulation study using (5) and the ‘confounding’ simulation study using (6), respectively:

(5) $$\lambda_{ij}\left(t\right)=\lambda_{0}\left(t\right)\exp\left(\beta_{0j}+\beta_{1}z_{ij}+\beta_{2}x_{ij}+\beta_{3}x_{ij}z_{ij}\right)$$

(6) $$\lambda_{ij}\left(t\right)=\lambda_{0}\left(t\right)\exp\left(\beta_{0j}+\beta_{1}z_{ij}+\beta_{2}x_{ij}+\beta_{3}x_{ij}z_{ij}+\beta_{4}y_{j}z_{ij}\right)$$

where the baseline hazards within each trial are proportional to the same common hazard function *λ*_0_(*t*), which is taken to be the exponential distribution with mean of 0.1. The fixed term *β*_0*j*_ for *j* = 1*,*2, …*,J* represents the change in the baseline hazard (from the reference *λ*_0_(*t*)) for each trial, where *β*_0*j*_ is sampled from a uniform distribution *U*(0*,*0*.*5), and *β*_1_, *β*_2_ and *β*_3_ are chosen to be fixed (the same for each trial) defining the treatment effect, adjustment factors and interaction, respectively. In model (6), the additional fixed term *β*_4_ defines the confounding factor in the ‘confounding’ simulation, which is chosen to be a positive constant. Each simulated dataset censored patients at 5 years if the event had not previously occurred.

Step 5 Steps 1–4 are repeated 1000 times to generate 1000 IPD meta‐analysis datasets for each simulation scenario of interest.
Step 6 To each 1000 meta‐analysis datasets generated, we fit either fixed‐effect or random‐effects models that either amalgamate within‐trial and across‐trial interactions (models (1) or (2)) or separate within‐trial and across‐trial interactions (models (3) or (4)). All models were fitted using maximum likelihood estimation via *coxme* in R, and in agreement with how the data were generated, in all models, we assumed that the covariate adjustment term was the same in each trial (i.e. that *β*_2*j*_ = *β*_2_); this also reduced potential non‐convergence issues.

Then to evaluate and compare the 1000 achieved parameter estimates from the different types of models, we look at the mean bias, mean standard error, mean squared error and coverage probability of 95% confidence intervals for each parameter estimate, with the performance of the estimates of the interaction terms of key interest for this article.

Our main focus is whether models (1) to (4) provide good estimates of the parameter *β*_3_ from the data generating models (5) and (6). *β*_3_ is the difference in treatment effect between two individuals who differ in *x*_*ij*_ by one‐unit and is thus informative toward stratified (personalized) treatment decisions. Therefore, it is important that one‐stage meta‐analysis models (1) to (4) provide unbiased estimates of *β*_3_, and so the simulation results in the following focus on comparing the estimates of *β*_*T*_, *β*_*W*_ and *β*_*A*_ from models (1) to (4) with the value of *β*_3_ used to generate the IPD.

### 3.1 Defining scenarios and parameter values

The simulation study focused on four key scenarios:

- ‘No confounding’ simulation study (model (5)): Binary variable (sex).
- ‘No confounding’ simulation study (model (5)): Continuous variable (age).
- ‘Confounding’ simulation study (model (6)): Binary variable (sex).
- ‘Confounding’ simulation study (model (6)): Continuous variable (age).

To generate the IPD meta‐analysis datasets for each scenario, the previous step by step process was used. To do this, we needed to define *β*_1_, *β*_2_, *β*_3_ and *β*_4_ and chose positive values for ease of use. To consider a reasonably large treatment effect, *β*_1_ was set to be 1 (i.e. a hazard ratio of 2.72, indicating the treatment is beneficial for a situation where the outcome is good, such as time to remission). *β*_2_ and *β*_3_ were defined to be 0.5 for the binary covariate (sex) and 0.01 for the continuous covariate (age). *β*_4_ was set to be 0 in the ‘no confounding’ simulation studies (as this parameter is not included in model (5)) and 0.75 in the ‘confounding’ simulations.

For each scenario, we also considered altering the number of trials and the number of observations per trial, that is *J* = 10 and *N* = 500 for the ‘large’ setting, and *J* = 5 and *N* = 250 for the ‘small’ setting. To explore the association between the scale of the covariate *x* and interaction effects, we also varied *V*_1_ and *V*_2_: for the binary case, *V*_1_ was chosen to be 0.4 or 0.2; and for the continuous case, *V*_1_ was set to be 20 or 10, and *V*_2_ was set to be 5 or 10. As mentioned previously, as *V*_1_ increases, the potential power of any across‐trial information will also increase. This is likely to be especially important in situations where *V*_1_ is also large relative to *V*_2_, such that the across‐trial information is potentially larger than the within‐study information 7.

In summary, our simulation study was repeated for each combination of *V*_1_, *V*_2_ and the sample size (*J* and *N*), for each of the ‘confounding’ and ‘no confounding’ situations, and for each of either a binary or a continuous covariate, and the results are now summarized in the following.

### 3.2 Results

#### 3.2.1 Binary covariate, no trial‐level confounding

Consider first the ‘no confounding’ simulations with the binary covariate (sex). Because there is no study‐level confounding, there is no unexplained heterogeneity across trials, and so the random‐effects models (2) and (4) are not considered here for brevity; however, their findings were almost identical to those from models (1) and (3).

A summary of the performance of the parameter estimates is shown in Table 1, for the different combinations of *V*_1_ and the sample size. In all settings, the true interaction between the log hazard ratio (treatment effect) and sex was 0.5, and so, if they reflected this, then the mean estimates of *β*_*T*_, *β*_*W*_ and *β*_*A*_ should be 0.5.

**Table 1:** The estimates of the treatment‐sex interaction effects in the simulations without trial level confounding.

		Model (1) (amalgamated interaction)				Model (3) (separated interactions)							
Size*	*V*_1_	Mean (SD)	Bias	MSE	Coverage	Mean (SD)		Bias		MSE		Coverage	
		*β*_*T*_	*β*_*T*_	*β*_*T*_	*β*_*T*_	*β*_*W*_	*β*_*A*_	*β*_*W*_	*β*_*A*_	*β*_*W*_	*β*_*A*_	*β*_*W*_	*β*_*A*_
Large	0.4	0.500 (0.072)	0	0.005	0.939	0.500 (0.082)	0.500 (0.164)	0	0	0.007	0.027	0.945	0.957
Large	0.2	0.501 (0.070)	0.001	0.005	0.946	0.502 (0.071)	0.490 (0.335)	0.002	−0.010	0.005	0.112	0.953	0.956
Small	0.4	0.494 (0.143)	−0.006	0.020	0.958	0.492 (0.153)	0.517 (0.484)	−0.008	0.017	0.023	0.235	0.964	0.945
Small	0.2	0.505 (0.138)	0.005	0.019	0.953	0.505 (0.143)	0.497 (0.841)	0.005	−0.003	0.020	0.707	0.948	0.959

N.B. In all settings, the true interaction between the log hazard ratio (treatment effect) and sex was 0.5, and so, if they reflected this, the mean estimates of *β*_*T*_, *β*_*W*_ and *β*_*A*_ should be 0.5.

* ‘Large’: *J* = 10 studies, *N* = 250 patients; ‘Small’: *J* = 5 studies, *N* = 250 patients.

MSE, mean‐square error; and SD, standard deviation of the 1000 parameter estimates.

In all settings, ${\hat{\beta }}_{T}$ from model (1) and ${\hat{\beta }}_{W}$ and ${\hat{\beta }}_{A}$ from model (3) were approximately unbiased estimates of the true treatment‐sex interaction effect, and coverage probabilities of their 95% confidence intervals were also very close to 0.95. For model (3), the mean squared errors of ${\hat{\beta }}_{W}$ were generally much smaller than those of ${\hat{\beta }}_{A}$. This highlights that the within trial interaction term usually has greater power than its across‐trial counterpart, and this difference becomes bigger as the number of studies or *V*_1_ decreases. However, ${\hat{\beta }}_{T}$ from model (1) has the smallest mean squared errors, as it is essentially a weighted combination of ${\hat{\beta }}_{W}$ and ${\hat{\beta }}_{A}$, and therefore, precision is improved, as indicated by the smaller standard deviations for ${\hat{\beta }}_{T}$ than ${\hat{\beta }}_{W}$ and ${\hat{\beta }}_{A}$.

#### 3.2.2 Continuous covariate, no trial‐level confounding

Table 2 summarizes the results for the continuous covariate (age) in the ‘no confounding’ scenarios. In all settings the true interaction between the log hazard ratio (treatment effect) and age was 0.01, and so, if they reflected this, the mean estimates of *β*_*T*_, *β*_*W*_ and *β*_*A*_ should be 0.01. The amalgamated effect, ${\hat{\beta }}_{T}$ from model (1) and the within‐ and across‐trial effects, ${\hat{\beta }}_{W}$ and ${\hat{\beta }}_{A}$, from model (3) were generally unbiased as they were close to 0.01 across all settings. The coverage in each setting was also very close to 0.95. As for the binary covariate, the amalgamated interaction ${\hat{\beta }}_{T}$ generally performs best because of larger precision (smaller standard errors).

**Table 2:** The estimates of the treatment‐age interaction effects in the simulations without trial level confounding.

			Model (1) (amalgamated interaction)				Model (3) (separated interactions)							
Size*	*V_1_*	*V_2_*	Mean(SD)	Bias	MSE	Coverage	Mean (SD)		Bias		MSE		Coverage	
			*β*_*T*_	*β*_*T*_	*β*_*T*_	*β*_*T*_	*β*_*W*_	*β*_*A*_	*β*_*W*_	*β*_*A*_	*β*_*W*_	*β*_*A*_	*β*_*W*_	*β*_*A*_
Large	20	10	0.010 (0.002)	0	<0.001	0.952	0.010 (0.003)	0.010 (0.004)	0	0	<0.001	<0.001	0.948	0.949
Large	20	5	0.010 (0.003)	0	<0.001	0.958	0.010 (0.006)	0.010 (0.003)	0	0	<0.001	<0.001	0.946	0.959
Large	10	10	0.010 (0.003)	0	<0.001	0.965	0.010 (0.003)	0.010 (0.007)	0	0	<0.001	<0.001	0.961	0.949
Large	10	5	0.010 (0.005)	0	<0.001	0.949	0.010 (0.006)	0.010 (0.007)	0	0	<0.001	<0.001	0.959	0.948
Small	20	10	0.010 (0.005)	0	<0.001	0.930	0.010 (0.007)	0.010 (0.009)	0	0	<0.001	<0.001	0.954	0.935
Small	20	5	0.010 (0.006)	0	<0.001	0.967	0.009 (0.013)	0.010 (0.008)	−0.001	0	<0.001	<0.001	0.953	0.960
Small	10	10	0.010 (0.006)	0	<0.001	0.945	0.010 (0.007)	0.011 (0.018)	0	0.001	<0.001	<0.001	0.947	0.953
Small	10	5	0.010 (0.010)	0	<0.001	0.949	0.010 (0.013)	0.010 (0.018)	0	0	<0.001	<0.001	0.949	0.952

N.B. In all settings, the true interaction between the log hazard ratio (treatment effect) and age was 0.01, and, so, if they reflected this, the mean estimates of *β*_*T*_, *β*_*W*_ and *β*_*A*_ should be 0.01.

* ‘Large’: *J* = 10 studies, *N* = 250 patients; ‘Small’: *J* = 5 studies, *N* = 250 patients.

MSE, mean square error; and SD, standard deviation of the 1000 parameter estimates.

In the scenarios with large sample size when using model (3), the standard error of the within‐ and across‐trial estimators were very similar, for example see the cases for *V*_1_ = 10*, V*_2_ = 5 or *V*_1_ = 20*, V*_2_ = 10. However, when *V*_1_ was large relative to *V*_2_, the standard error of ${\hat{\beta }}_{W}$ appeared slightly larger than ${\hat{\beta }}_{A}$. For example, in the ‘large’ setting and *V*_1_ = 20 and *V*_2_ = 5, the standard deviation of ${\hat{\beta }}_{W}$ was 0.006 whilst the standard deviation of ${\hat{\beta }}_{A}$ was 0.003. Conversely, when *V*_1_ was small or similar relative to *V*_2_, the standard error of ${\hat{\beta }}_{W}$ was smaller than ${\hat{\beta }}_{A}$. For example, in the ‘large’ setting given *V*_1_ = 10 and *V*_2_ = 10, the standard deviation of ${\hat{\beta }}_{W}$ was 0.003 whilst ${\hat{\beta }}_{A}$ was 0.007.

These findings confirm previous work 7: the power to detect the patient‐level interaction effects using ${\hat{\beta }}_{W}$ increases when *V*_2_ increases, and when using ${\hat{\beta }}_{A}$ it increases when *V*_1_ increases. For the simulations with small sample size, findings were similar except standard errors were of a larger magnitude throughout.

#### 3.2.3 Binary covariate, trial‐level confounding

Consider now the situation of a binary covariate when there is unknown trial‐level confounding (because of treatment dose, relating to *y_j_* in step 3(b) of the process used to simulate the IPD), and thus residual between‐study heterogeneity. The simulation results are summarized in Table 3.

**Table 3:** The estimators of the treatment‐sex interaction effects in the simulated data considering trial‐level treatment confounding.

Size*	*V*_1_	Model	Mean (SD)		Bias	MSE	Coverage	Model	Mean (SD)			Bias		MSE		Coverage	
			*β*_*T*_	*τ*	*β*_*T*_	*β*_*T*_	*β*_*T*_		*β*_*W*_	*β*_*A*_	*τ*	*β*_*W*_	*β*_*A*_	*β*_*W*_	*β*_*A*_	*β*_*W*_	*β*_*A*_
Large	0.4	(1)	0.721 (0.091)		0.221	0.057	0.192	(3)	0.495 (0.078)	1.970 (0.320)		−0.005	1.470	0.006	2.263	0.944	0.003
Large	0.4	(2)	0.528 (0.079)	0.125(0.034)	0.028	0.007	0.927	(4)	0.499 (0.078)	1.967 (0.317)	0.028 (0.019)	−0.001	1.467	0.006	2.252	0.947	0.008
Large	0.2	(1)	0.599 (0.076)		0.099	0.016	0.696	(3)	0.496 (0.071)	3.400 (0.638)		−0.004	2.900	0.005	8.816	0.943	0.000
Large	0.2	(2)	0.510 (0.071)	0.127(0.034)	0.010	0.005	0.947	(4)	0.499 (0.071)	3.401 (0.634)	0.028 (0.017)	−0.001	2.901	0.005	8.815	0.946	0.006
Small	0.4	(1)	0.694 (0.176)		0.194	0.069	0.683	(3)	0.490 (0.162)	1.977 (0.749)		−0.010	1.477	0.026	2.741	0.949	0.107
Small	0.4	(2)	0.547 (0.168)	0.105(0.069)	0.047	0.030	0.934	(4)	0.492 (0.162)	1.978 (0.747)	0.019 (0.028)	−0.008	1.478	0.026	2.742	0.949	0.196
Small	0.2	(1)	0.597 (0.139)		0.097	0.029	0.897	(3)	0.501 (0.135)	3.463 (1.543)		0.001	2.963	0.018	11.159	0.962	0.086
Small	0.2	(2)	0.524 (0.136)	0.111(0.065)	0.024	0.019	0.958	(4)	0.502 (0.136)	3.470 (1.537)	0.018 (0.027)	0.002	2.970	0.018	11.184	0.963	0.157

N.B. In all settings, the true interaction between the log hazard ratio (treatment effect) and sex was 0.5, and so, if they reflected this, the mean estimates of *β*_*T*_, *β*_*W*_ and *β*_*A*_ should be 0.5.

* ‘Large’: *J* = 10 studies, *N* = 250 patients; ‘Small’: *J* = 5 studies, *N* = 250 patients.

MSE, mean square error; and SD, standard deviation of the 1000 parameter estimates.

Consider the fixed and random‐effects models (3) and (4), which treated the within and across‐trial interaction terms separately. The patient level interaction estimators, ${\hat{\beta }}_{W}$, were still approximately unbiased for all settings as they were very close to the true value, 0.5. However, because of the unaccounted for trial‐level confounder of dose in models (3) and (4), ${\hat{\beta }}_{A}$ was now biased in every setting. For example, given *V*_1_ = 0*.*2 and the ‘small’ sample setting, the mean of ${\hat{\beta }}_{W}$ was 0*.*502 from the random‐effects model (4) and so close to the truth, whereas the mean of the across‐trial interaction estimator, ${\hat{\beta }}_{A}$, was 3*.*47 and so had serious upward bias. The stark difference between the estimators of ${\hat{\beta }}_{W}$ and ${\hat{\beta }}_{A}$ demonstrates the impact of ecological bias on ${\hat{\beta }}_{A}$, because of the unaccounted trial‐level confounder of dose. Interestingly, the bias in ${\hat{\beta }}_{A}$ was not improved when using the random‐effects model (4) rather than the fixed‐effect model (3). There was also very poor MSE and coverage of ${\hat{\beta }}_{A}$ because of the presence of ecological bias, whereas MSE was small and coverage acceptable for ${\hat{\beta }}_{W}$.

Models (1) and (2) also gave estimates of *β*_*T*_ that were upwardly biased compared with 0.5. The random‐effects model (2) performed better in terms of the coverage being closer to 0.95, but ${\hat{\beta }}_{T}$ was still upwardly biased in most settings because of amalgamating the unbiased within trial interaction with the upwardly biased across‐trial interaction. For example, given the ‘large’ setting and *V*_1_ = 0*.*4, the mean estimate of *β_T_* was 0.528 (coverage = 0.927) for the random‐effects model and 0.721 (coverage = 0.192) for the fixed‐effect model, which are both above the true value of 0.5.

In summary, the bias, MSE and standard error are vastly superior for ${\hat{\beta }}_{W}$ in these settings, and there are serious issues with ${\hat{\beta }}_{A}$.

#### 3.2.4 Continuous covariate, trial‐level confounding

The results for a continuous covariate in the setting of trial‐level confounding are now summarized in Table 4. In all settings for both models (3) and (4), the mean and coverage of ${\hat{\beta }}_{W}$ were close to 0.01 and 0.95, respectively, indicating excellent performance of the within trial interaction. On the contrary, the ${\hat{\beta }}_{A}$ estimates from the two models were upwardly biased with coverage much lower than 0.95, highlighting again the impact of ecological bias because of the omission of the trial‐level confounder of dose used to generate the IPD in these settings (see step 3(b) of the simulation set‐up).

**Table 4:** The estimates of the treatment‐age interaction effects in the simulated data considering trial‐level treatment confounding.

Size*	*V*_1_	*V*_2_	Model	Mean (SD)		Bias	MSE	Coverage	Model	Mean (SD)			Bias		MSE		Coverage	
				*β*_*T*_	*τ*	*β*_*T*_	*β*_*T*_	*β*_*T*_		*β*_*W*_	*β*_*A*_	*τ*	*β*_*W*_	*β*_*A*_	*β*_*W*_	*β*_*A*_	*β*_*W*_	*β*_*A*_
Large	20	10	(1)	0.023 (0.003)		0.013	<0.001	0.018	(3)	0.010 (0.003)	0.040 (0.006)		0	0.030	<0.001	0.001	0.958	0.003
Large	20	10	(2)	0.013 (0.003)	0.112 (0.040)	0.003	<0.001	0.848	(4)	0.010 (0.003)	0.041 (0.006)	0.027(0.019)	0	0.031	<0.001	0.001	0.958	0.010
Large	20	5	(1)	0.032 (0.004)		0.022	<0.001	0.003	(3)	0.010 (0.006)	0.039 (0.006)		0	0.029	<0.001	0.001	0.965	0.002
Large	20	5	(2)	0.021(0.006)	0.068 (0.043)	0.011	<0.001	0.418	(4)	0.010 (0.006)	0.039 (0.006)	0.025(0.018)	0	0.029	<0.001	0.001	0.964	0.005
Large	10	10	(1)	0.020 (0.003)		0.010	<0.001	0.131	(3)	0.010 (0.003)	0.068 (0.012)		0	0.058	<0.001	0.003	0.949	0.003
Large	10	10	(2)	0.011 (0.003)	0.121 (0.037)	0.001	<0.001	0.927	(4)	0.010 (0.003)	0.068 (0.012)	0.026(0.018)	0	0.058	<0.001	0.004	0.946	0.005
Large	10	5	(1)	0.035 (0.006)		0.025	0.001	0.018	(3)	0.010 (0.007)	0.06 (0.012)		0	0.058	<0.001	0.003	0.945	0.001
Large	10	5	(2)	0.015 (0.007)	0.109 (0.041)	0.005	<0.001	0.826	(4)	0.010 (0.007)	0.068 (0.012)	0.026(0.019)	0	0.058	<0.001	0.003	0.945	0.004
Small	20	10	(1)	0.022 (0.007)		0.012	<0.001	0.38	(3)	0.010 (0.007)	0.041 (0.017)		0	0.031	<0.001	0.001	0.942	0.121
Small	20	10	(2)	0.015 (0.008)	0.085 (0.078)	0.005	<0.001	0.778	(4)	0.010 (0.007)	0.041 (0.017	0.015(0.026)	0	0.031	<0.001	0.001	0.943	0.184
Small	20	5	(1)	0.030 (0.010)		0.020	0.001	0.173	(3)	0.010 (0.013)	0.039 (0.017)		0	0.029	<0.001	0.001	0.939	0.115
Small	20	5	(2)	0.024 (0.012)	0.050 (0.073)	0.014	<0.001	0.464	(4)	0.010 (0.013)	0.039 (0.017)	0.017(0.028)	0	0.029	<0.001	0.001	0.939	0.185
Small	10	10	(1)	0.019 (0.007)		0.009	<0.001	0.665	(3)	0.010 (0.006)	0.071 (0.032)		0	0.061	<0.001	0.005	0.953	0.103
Small	10	10	(2)	0.012 (0.007)	0.106 (0.069)	0.002	<0.001	0.917	(4)	0.010 (0.006)	0.071 (0.032)	0.019(0.030)	0	0.061	<0.001	0.005	0.952	0.179
Small	10	5	(1)	0.032 (0.012)		0.022	0.001	0.354	(3)	0.009 (0.013)	0.069 (0.031)		−0.001	0.059	<0.001	0.004	0.961	0.109
Small	10	5	(2)	0.019 (0.014)	0.086 (0.075)	0.009	<0.001	0.808	(4)	0.009 (0.013)	0.069 (0.031	0.016(0.027)	−0.001	0.059	<0.001	0.004	0.961	0.185

In all settings, the true interaction between the log hazard ratio (treatment effect) and age was 0.01, and so, if they reflected this, the mean estimates of *β*_*T*_, *β*_*W*_ and *β*_*A*_ should be 0.01.

* ‘Large’: *J* = 10 studies, *N* = 250 patients; ‘Small’: *J* = 5 studies, *N* = 250 patients.

MSE, mean square error; and SD, standard deviation of the 1000 parameter estimates.

When using models (1) and (2), the results show that ${\hat{\beta }}_{T}$ also had poor performance in terms of bias and coverage, especially when the fixed‐effect model (1) was utilized. Although the standard error of ${\hat{\beta }}_{T}$ was sometimes smaller than ${\hat{\beta }}_{W}$, this only arose by utilizing the biased ${\hat{\beta }}_{A}$. As noted elsewhere, the gain in standard error comes at the expense of bias and poorer coverage 12, 14, 23.

### 3.3 Summary of simulation findings

In conclusion, our simulation study has demonstrated that to understand how a patient‐level covariate interacts with treatment effect, it is generally better to examine *β*_*W*_ rather than either the trial‐level interaction effect, *β*_*A*_, or the amalgamated interaction effect, *β*_*T*_. Although ${\hat{\beta }}_{T}$ performs best when there is no trial‐level confounding (because of larger precision and smaller MSE), its performance deteriorates considerably when trial‐level confounding exists as its estimate and coverage are then severely affected by ecological bias, which may produce misleading conclusions. The magnitude of such bias is worse when using a fixed‐effect model. Although it is still non‐negligible in a random‐effects model, the inclusion of residual between‐trial heterogeneity reduces the power of the across‐trial information, such that it has less weight toward ${\hat{\beta }}_{T}$ than in a fixed‐effect model. In contrast, the performance of ${\hat{\beta }}_{W}$ remains excellent in all situations considered, as it separates the within‐trial information from the across‐trial information. Finally, we note that an alternative two‐stage approach to obtain the interaction estimate in each trial separately, followed by a traditional fixed‐effect or random‐effects model, gave almost identical results to those shown for ${\hat{\beta }}_{W}$ from one‐stage models (3) or (4).

## 4 Application to an IPD meta‐analysis in epilepsy

Epilepsy is one of the most common neurological disorders threatening 65 million people throughout the world 49. Previous researchers conducted an IPD meta‐analysis of 1225 patients from five randomized controlled trials to compare the effects of two antiepileptic drugs, Sodium Valproate (SV, drug = 1) and Carbamazepine (CBZ, drug = 0), when used as monotherapy in patients with partial onset seizures or generalized onset seizures 50, 51, 52. Here, we focus on the treatment effect (SV versus CBZ) on the outcome of time to 12 month remission, in relation to three patient‐level covariates: age at randomization (in years), type of epilepsy (generalized or partial) and the log number of seizures in 6 months before randomization. In a previous analysis of this data, Tudur Smith *et al*. examine interactions between the treatment effects and these three covariates 50 using one‐stage models; however, these only had an amalgamated interaction term. Hence, here, we examine if separation of within‐trial and across‐trial interaction alters the original conclusions.

For each covariate separately, we used maximum likelihood (via the *coxme* module in R) to estimate models (1) and (2), which amalgamate within‐trial and across‐trial interactions, and models (3) and (4), which separate out within‐trial and across‐trial interactions. There were sometimes estimation problems when a separate covariate adjustment term was used in each trial, and so the results shown in Table 5 are from models (1) to (4) but with *β*_2*j*_ = *β*_2_, as this resolved any non‐convergence during model estimation. In situations where convergence was possible with separate *β*_2*j*_ terms, the results and conclusions were very similar to those shown. We also refitted all our models using the Poisson regression approach of Crowther *et al*. 37, and results were again very similar. We focus now only on the results for the interaction estimates (Table 5). As noted for the simulation study, an alternative two‐stage approach gives almost identical results to those shown for ${\hat{\beta }}_{W}$ from one‐stage models (3) or (4).

**Table 5:** Summary of the treatment‐covariate effect estimates in the epilepsy data for the outcome of time to 12 months remission.

Covariate	Model	${\hat{\beta }}_{T}$ (SE)	CI of ${\hat{\beta }}_{T}$	Model	${\hat{\beta }}_{W}$ (SE)	${\hat{\beta }}_{A}$ (SE)	CI of ${\hat{\beta }}_{W}$	CI of ${\hat{\beta }}_{A}$
Age at randomization	(1)	−0.011* (0.004)	−0.019 to −0.003	(3)	−0.007 (0.006)	−0.013* (0.005)	−0.019 to 0.005	−0.023 to −0.003
	(2)	−0.011* (0.004)	−0.019 to −0.003	(4)	−0.007 (0.006)	−0.013* (0.005)	−0.019 to 0.005	−0.023 to −0.003
Epilepsy type	(1)	−0.128 (0.147)	−0.416 to 0.160	(3)	−0.026 (0.168)	−0.467 (0.307)	−0.355 to 0.303	−1.069 to 0.135
	(2)	−0.090 (0.156)	−0.396 to 0.216	(4)	−0.025 (0.168)	−0.479 (0.376)	−0.354 to 0.304	−1.216 to 0.258
Log number of seizures	(1)	−0.025 (0.056)	−0.135 to 0.085	(3)	−0.014 (0.058)	−0.100 (0.122)	−0.128 to 0.100	−0.339 to 0.139
	(2)	−0.020 (0.057)	−0.132 to 0.092	(4)	−0.013 (0.058)	−0.105 (0.142)	−0.127 to 0.101	−0.383 to 0.173

* *p*‐value < 0.01.

SE, standard error of the parameter estimate; CI, confidence interval.

There was no evidence that either epilepsy type or the log number of seizures were modifiers of the treatment effect for any models (*p >* 0*.*1). However, for both covariates, the amalgamated estimator, ${\hat{\beta }}_{T}$, was larger in absolute magnitude than the patient‐level estimator, ${\hat{\beta }}_{W}$, suggesting that ecological bias may be present. For example, in the random‐effects model for epilepsy type, ${\hat{\beta }}_{T}$ was −0*.*09 (standard error (SE) = 0.156) and much larger than the ${\hat{\beta }}_{W}$ value of −0*.*025 (SE = 0.058). This was due to ${\hat{\beta }}_{T}$ being an amalgamation of ${\hat{\beta }}_{W}$ with an extremely large ${\hat{\beta }}_{A}$ = −0*.*479 (SE = 0.376). Interestingly, one of the five trials (Mattson) only had partial epilepsy type patients and thus provides some across‐trials information but no within‐trial information toward this ${\hat{\beta }}_{T}$.

The findings were even more dramatic for age, as statistical significance at the 5% level was different for ${\hat{\beta }}_{T}$ and ${\hat{\beta }}_{W}$. The within‐trial effect, ${\hat{\beta }}_{W}$, was not statistically significant ( ${\hat{\beta }}_{W}$ = −0*.*007, 95% CI: −0.019 to 0.005, *p* = 0.219) whereas the amalgamated effect estimator was larger and statistically significant ( ${\hat{\beta }}_{T}$ =  −0*.*011, 95% CI: −0.019 to −0.003) (*p* = 0.004). Again, the difference arises due to ${\hat{\beta }}_{T}$ amalgamating ${\hat{\beta }}_{W}$ with ${\hat{\beta }}_{A}$, which increases precision but at the expense of ${\hat{\beta }}_{A}$ introducing potential ecological bias (study‐level confounding), because ${\hat{\beta }}_{W}$ is about half the size of ${\hat{\beta }}_{A}$ (Table 5).

The analysis of age was extended to replicate the original analysis of Tudur Smith *et al*., which included additional adjustment terms for epilepsy type and log number of seizures. The findings remained similar: the within‐trial effect, ${\hat{\beta }}_{W}$, was not statistically significant ( ${\hat{\beta }}_{W}$ = −0.006, 95% CI: −0.017 to 0.005, *p* = 0.298), whereas the amalgamated effect estimate was statistically significant ( ${\hat{\beta }}_{T}$ = − 0.008, 95% CI: −0.016 to −0.001) (*p* = 0.024). Thus, based on the within‐trial interaction alone, there is not strong evidence that age is a moderator of treatment effect, and further research is recommended, which adds new insight on previous analyses of this data 50, 51, 52.

## 5 Discussion

Individual participant data meta‐analyses are increasingly prominent for time‐to‐event data, as the availability of IPD often allows a longer follow‐up time and more sophisticated modelling than an aggregate data meta‐analysis. In particular, IPD meta‐analyses of cancer studies are usually time‐to‐event, and there is enormous interest in their use for examining whether biomarkers are treatment effect modifiers to inform precision oncology 53 and for deriving absolute risk prediction models 40. One‐stage IPD models are often preferred, as this produces all meta‐analysis results in a single analysis and is potentially more flexible, for example in regard modelling the baseline hazard, non‐proportional hazards and non‐linear trends, than a two‐stage approach. It is therefore critical that researchers use the correct one‐stage IPD modelling approach.

In this article, we compared, through simulations and an applied example, different specifications of a *one‐stage* IPD meta‐analysis model of time‐to‐event data where the goal is to estimate a treatment‐covariate interaction. Our findings agree with previous work and simulations for continuous and binary outcomes 9, 23: it is crucial to separate within‐trial and across‐trial interactions, to avoid ecological bias caused by unexplained trial‐level confounding 8, 54, 55. Otherwise, clinical conclusions about interactions may be driven by ecological, trial‐level information rather than solely within‐trial information at the individual‐level. This is especially important when the power of any across‐trial information is comparable with that for within‐trial information, which occurs when the variation across‐trials in the mean covariate values is similar to, or bigger than, the variation in individual covariate values 7. The consequences of amalgamating within‐trial and across‐trial interactions may be substantial with false predictors of treatment effect being wrongly identified as important, or conversely genuine predictors of treatment effect being missed or discarded prematurely 1. Our epilepsy example demonstrates how the magnitude of treatment effect modification for age and its statistical significance depends heavily on whether within‐trial associations are amalgamated or separated from across‐trial associations; in particular, separating within‐trial and across‐trial information leads to less dramatic clinical and statistical conclusions.

Although our simulations show that amalgamating within‐trial and across‐trial associations can improve precision of treatment‐covariate interactions, this is not an adequate justification for doing so given the clear, adverse consequences on bias and coverage when trial‐level confounding exists. In our opinion, gain in precision must not be made at the expense of potential bias and poor coverage. In situations of trial‐level confounding, our simulations show that bias and inappropriate coverage of the amalgamated interaction occur regardless of whether it is estimated in a fixed‐effect or random‐effects setting, although the impact is far worse when using fixed‐effect models.

Simmonds *et al*. reviewed a sample of IPD meta‐analyses of randomized trials published from 2008 to 2014 30 and state that: ‘In one‐stage analyses, most papers reported including covariates in the one‐stage regression model (21 reviews), although exactly how this was carried out was rarely reported’. This is concerning, as we would expect IPD publications to state that ecological bias was avoided by separating out within‐trial and across‐trial interactions, if indeed it had been carried out; hence, the absence of such reporting suggests interactions were (perhaps unknowingly) based on amalgamating within‐trial and across‐trial associations. Further empirical evidence would be welcome.

Our recommendation to focus only on *β*_*W*_ echoes previous calls by Thompson and colleagues, who state that ‘within‐study and between‐study information for interactions need to be distinguished’ 14 and ‘in general, we would suggest that the estimated relations between the extent of treatment benefit and patients' characteristics are derived only from within‐trial information, so that confounding because of differences across trials is avoided’ 4. In situations where IPD are limited and most information comes from across trials (for example, when IPD are not available for all studies 9, 20, 23 or the variation in particular covariate values within trials is small or even zero), again, this does not provide credence for making recommendations based on the trial‐level information because of the aforementioned issues. At best, meta‐regression analyses using the trial‐level should only be viewed as exploratory when the aim is to identify individual‐level associations and should not inform clinical recommendations. However, we recognize that others may not agree with these recommendations; for example, models have been proposed for combining across‐study and within‐study interactions when a mixture of IPD and AD are available 56, 57.

In our simulations, we did not consider the extra complexity of potential confounding within‐trials when examining whether a particular factor interacts with treatment. Furthermore, even when the analysis of a particular factor produces a *β*_*W*_ that is statistically and clinical important, there may still be debate about whether the factor is a genuine causal modifier of treatment effect. Sun *et al*.^58^ provide guidance for identifying whether differences in subgroups are believable, and this includes consideration of biological plausibility.

If one is interested in the overall effect for particular subgroups (e.g. men and women), then a separate one‐stage model could be fitted for each (thus avoiding interaction terms). However, before making statements about differences between subgroups, it is crucial to test/quantify their difference using ${\hat{\beta }}_{W}$, for which our one‐stage models that separate out within‐trial and across‐trial interactions are needed. Of course, a traditional two‐stage meta‐analysis of interaction estimates would also avoid ecological bias and, in most situations, will give a summary meta‐analysis result very similar to ${\hat{\beta }}_{W}$ from our one‐stage models that separate within‐trial and across‐trial interactions. However, especially in situations with small numbers of events, the more exact likelihood for the one‐stage approach may give better statistical properties than the two‐stage approach, for which the assumption of normally distributed estimates and known within‐study variances may be inappropriate 16. Ecological bias may also affect a two‐stage approach where the interactions are jointly synthesized with other parameter estimates (such as intercepts); for further discussion, see Riley *et al*.^59^.

In conclusion, where one‐stage models are to be used to examine potential treatment‐covariate interaction, researchers should pre‐specify in their protocol that they will separate out within‐trial and across‐trial interactions. Furthermore, recommendations about predictors of differential treatment effect should only be based on within‐trial interactions, to avoid potentially erroneous implications for clinical practice that can arise when within‐trial and across‐trial information is amalgamated.

## Funding

Danielle Burke is funded by an NIHR School for Primary Care Research Post‐Doctoral Fellowship. The views expressed are those of the author(s) and not necessarily those of the NHS, the NIHR or the Department of Health. We wish to thank the Epilepsy Monotherapy Trialists' Group for making their individual patient data available. Catrin Tudur Smith received funding from the MRC Network of Hubs for Trials Methodology Research (MR/K025635/1).

## Supporting information

Supporting info item
