# Supplementary material for: One‐stage individual participant data meta‐analysis models: estimation of treatment‐covariate interactions must avoid ecological bias by separating out within‐trial and across‐trial information
Source: Stat Med. 2016 Dec 1;36(5):772–89. doi: 10.1002/sim.7171 (PMC5299543; doi:10.1002/sim.7171)
Supplement: Supplementary file 1 — Supporting info item [file SIM-36-772-s001.pdf]

Supplementary material: Full epilepsy example results

Outcome	Covariate	Model	Parameter Estimate (s.e.) B_{drug}	β_{cov}	β_T	τ	Model	Parameter Estimate (s.e.) B_{drug}	β_{cov}	β_W	β_A	τ
Time to 12 month remission	Age at randomisation	(1)	0.199<br>(0.129)	0.008<br>(0.003)	-0.011***<br>(0.004)	0.004	(3)	0.269*<br>(0.158)	0.006<br>(0.004)	-0.007<br>(0.006)	-0.013***<br>(0.005)	0.004
		(2)	0.199<br>(0.129)	0.008**<br>(0.003)	-0.011***<br>(0.004)		(4)	0.269*<br>(0.158)	0.006<br>(0.004)	-0.007<br>(0.006)	-0.013***<br>(0.005)	
	Epilepsy type	(1)	-0.035<br>(0.113)	-0.238**<br>(0.110)	-0.128<br>(0.147)		(3)	0.168<br>(0.197)	-0.287**<br>(0.118)	-0.026<br>(0.168)	-0.467<br>(0.307)	
		(2)	-0.039<br>(0.132)	-0.256**<br>(0.113)	-0.090<br>(0.156)	0.136	(4)	0.186<br>(0.239)	-0.287**<br>(0.118)	-0.025<br>(0.168)	-0.479<br>(0.376)	0.106
	Log number of seizures	(1)	-0.037<br>(0.121)	-0.166***<br>(0.040)	-0.025<br>(0.056)		(3)	0.112<br>(0.245)	-0.171***<br>(0.041)	-0.014<br>(0.058)	-0.100<br>(0.122)	
		(2)	-0.030<br>(0.131)	-0.168***<br>(0.040)	-0.020<br>(0.057)	0.104	(4)	0.134<br>(0.285)	-0.171***<br>(0.041)	-0.013<br>(0.058)	-0.105<br>(0.142)	0.096

N.B. * for P-value< 0.1, ** for P-value< 0.05 and *** for P-value<0.01

For Peer Review
